# Molecular Characterization of Motile Serovars of *Salmonella enterica* from Breeder and Commercial Broiler Poultry Farms in Bangladesh

**DOI:** 10.1371/journal.pone.0057811

**Published:** 2013-03-06

**Authors:** Himel Barua, Paritosh K. Biswas, Katharina E. P. Olsen, Subrata K. Shil, Jens P. Christensen

**Affiliations:** 1 Department of Veterinary Disease Biology, Faculty of Health and Medical Sciences, University of Copenhagen, Frederiksberg, Copenhagen, Denmark; 2 Department of Microbiology, Faculty of Veterinary Medicine, Chittagong Veterinary and Animal Sciences University, Chittagong, Bangladesh; 3 The National Reference Laboratory for Enteropathogenic Bacteria, Department of Microbiology and Infection Control, Statens Serum Institut, Copenhagen, Denmark; 4 Department of Anatomy and Histology, Faculty of Veterinary Medicine, Chittagong Veterinary and Animal Sciences University, Chittagong, Bangladesh; Indian Institute of Science, India

## Abstract

Contaminated poultry and poultry products are a major source of motile Salmonellae for human salmonellosis worldwide. Local circulation of any motile *Salmonella* serovar in poultry has a wider public health impact beyond its source of origin for being dispersed elsewhere through poultry trades or human travels. To investigate the status of motile *Salmonella* serovars in breeder farms in Bangladesh, multiple flocks of two breeder farms were observed for a period of six months. In addition, a cross-sectional survey was carried out to determine the prevalence and serovar distribution of motile *Salmonella* by randomly selecting 100 commercial broiler poultry farms. Five pooled faecal samples representing an entire housed flock of breeders or broilers were screened for presence of motile *Salmonella* following conventional bacteriological procedures. The *Salmonella* isolates obtained were subsequently serotyped, and characterized by plasmid profiling and pulsed-field gel electrophoresis (PFGE). The results revealed that both the breeder farms were positive with three *Salmonella* serovars: *S*. Virchow, *S*. Paratyphi B var Java (*S*. Java) and *S*. Enteritidis. Eleven of the 100 broiler farms investigated were positive for motile *Salmonella*, giving a farm-level prevalence of 11% (95% confidence interval 5–17%). *S*. Virchow and *S.* Kentucky were the two predominant serovars isolated from the broiler farms. The PFGE genotyping demonstrated that the isolates belonging to the same serovars were closely related due to variation in only 1–4 bands. All the *S.* Virchow and *S.* Java isolates, irrespective of breeder or broiler farm origin, were plasmid-free, except for one *S.* Virchow isolate from a broiler farm that harboured a 9.7 kb-sized plasmid. The *S.* Kentucky isolates belonged to three plasmid profiles having plasmids of four different sizes, ranging from 2.7 to 109 kb. This is the first report of any motile *Salmonella* serovars from breeder and commercial broiler poultry farms in Bangladesh.

## Introduction

*Salmonella* is a major food-borne pathogen, frequently associated with human gastroenteritis throughout the world. The global burden of human gastroenteritis due to *Salmonella* has been estimated 93.8 million cases, resulting in 155,000 deaths each year [1]. Poultry is considered a major reservoir for many non-host specific motile serovars of *Salmonella*, and often human infection is attributed to consumption of contaminated poultry products, such as eggs and meats [2]. There are >2500 serovars of *Salmonella enterica* [3] and all motile serovars of them are zoonotic with variable infection intensities. Although *Salmonella enterica* subsp. *enterica* serovar Enteritidis (*S.* Enteritidis) and *S*. Typhimurium are commonly isolated from poultry and often cause human infections [4]–[6], other poultry-originating emerging serovars are also reported [7], [8]. Cross border or intercontinental transmission may occur as observed with the circulating *S*. Kentucky ST198 clone [9]. Any emergence of a new zoonotic serovar of *Salmonella* in poultry in any locality therefore not only has local consequences but may also be a global public health concern because it can be transmitted to any parts of the world through poultry trades or human travels [9]–[12].

The four FAO-defined [13] poultry production systems, namely – industrial and integrated (sector 1), large-scale commercial (sector 2), small-scale commercial (sector 3) and backyard (sector 4) are all seen in Bangladesh. Within the commercial sector, the small-scale commercial production system predominates, to which day-old chicks are supplied from approximately 40–50 breeder farms. In parallel, there are a few public owned breeder farms which produce a cross-bred (F1 generation) chick, locally called “Sonali” (Fayoumi (hen) × Rhode Island Red (cock)) which are supplied to the smallholders at a subsidized rate as part of the poverty alleviation programme. *Salmonella* infected breeder farms can be sustained sources for vertically transmitting the organism to commercial or smallholders’ farms, and consequently to humans through a contaminated food chain [14]. However, in the absence of any structured surveillance for *Salmonella* in Bangladesh, *Salmonella* status in the different poultry production systems is largely unknown. Recently a survey of commercial layer poultry farms was conducted, the results of which demonstrated the presence of only one serovar - *S*. Kentucky, indicating a wide dissemination of this serovar in layer poultry population in Bangladesh [15]. Whether breeder farms play any role in its perpetuation, or are harbouring other zoonotic serovars, to transmit to commercial farms remain unexplored. To prevent local and international spread of zoonotic *Salmonella* attributable to poultry products, the epidemiology of these infections has to be investigated nationally in order to suggest future control programmes. Here, the motile *Salmonella* serovars circulating in breeders and broilers in Bangladesh and their molecular characteristics are described.

## Materials and Methods

### Ethics Statement

No specific permits were required for the described field studies. Verbal consent from the authority of each breeder- and commercial broiler poultry farm was taken during collecting the faecal samples.

### Study Population

Dhaka, the capital city and Chittagong, the second largest city in Bangladesh are the two major poultry hubs [16] due to the better marketing facilities. Recently, from these two districts Chittagong was chosen by lottery to carry out a survey to estimate the prevalence of motile *Salmonella* in commercial layer poultry farms [15]. To investigate the distribution of motile *Salmonella* serovars in breeder- and commercial broiler poultry farms in Bangladesh, we therefore selected the same district, Chittagong. Two breeder farms – one representing the commercial and the other the public poultry sector, hereafter referred to as “Farm A” and “Farm B”, respectively, were enrolled for the present investigation. Each farm had eight flocks. A flock size in “Farm A” ranged from 5000 to 15000, and 250 to 5500 in “Farm B”. The age of the birds belonging to the sampled flocks on the day of first sampling ranged from 5 to 57 weeks in “Farm A” and 4 to 44 weeks in “Farm B”. Flocks were sampled monthly for a period of six months.

Additionally, from the list of all the commercial broiler poultry farms in the district of Chittagong, kept at the District Livestock Office, we randomly selected 100 farms by generating random numbers in computer to undertake a cross-sectional survey for the prevalence of motile *Salmonella*. All the selected commercial broiler poultry farms were of the FAO-defined production system 3, the small-scale commercial type.

### Sample Collection

We observed the selected flocks of two breeder farms for a period of six months – from May to October 2010 while the cross-sectional survey in commercial broiler poultry farms was conducted from May to July 2010. Each flock of the two breeder farms was visited monthly to collect pooled faecal samples. All the broiler farms were single-housed and faecal samples were therefore collected from a single flock. Faecal samples were collected from floor covered with litter. The use of litter materials varied according to farms – in breeder farms, rice husk was used while in the broiler farms either saw dust or rice husk was employed. For a flock belonging to any of the breeder or commercial broiler poultry farms, five naturally pooled faecal samples from five different locations of the house were collected; each sample consisted of ∼30 cross-sectional pinches of faeces mixed with litter for obtaining a total weight of approximately 250 g. Each pooled sample was collected by gloved hands, placed separately into a sterile plastic bag, properly labelled and transported to the microbiology laboratory, Chittagong Veterinary and Animal Sciences University (ML-CVASU), Bangladesh at environmental temperature. On arrival at ML-CVASU, the samples were stored at 5°C until analyzed.

### Isolation and Identification of *Salmonella*

Conventional bacteriological procedures were followed for isolation and identification of *Salmonella* from the faecal samples. Briefly, 200 g of faecal samples were mixed homogenously with 200 ml of buffered peptone water (BPW) (Oxoid Ltd., England) to make slurry. Fifty gram from this slurry was transferred to 200 ml BPW and incubated at 37°C for 18±1 hours, followed by selective enrichment in Rappaport-Vassiliadis (RV) broth (Scharlau Chemie S. A., EU) for 24 to 48 hours at 42°C. From this cultured broth, 10 µl was streaked onto brilliant green (BG) agar (Oxoid Ltd., England) surface and incubated overnight at 37°C. Any suspected *Salmonella* colony on BG agar was verified and identified with standard biochemical tests applied for identification of *Salmonella*. A breeder flock or a broiler farm was considered presumptively *Salmonella*-positive when *Salmonella* was isolated from ≥1 of the five collected samples. All *Salmonella* isolates from the breeder farms and one isolate per positive broiler farm were stored at −80°C and shipped later to the Department of Veterinary Disease Biology, University of Copenhagen (KU-DVDB) using Stuart’s transport medium (Oxoid Ltd., England) at environmental temperature by a professional courier service.

At KU-DVDB, the isolates were screened further for confirmation of motile *Salmonella* by growing the bacteria on Luria Bertani (LB) broth (Difco, USA) at 37°C and subsequently transferring 100 µl of the overnight culture (divided into 3 separate drops) on to novobiocin (Oxoid Ltd., England) supplemented Modified Semisolid Rappaport Vassiliadis (MSRV) agar (Oxoid Ltd., England) and incubated for 24 hours at 41.5°C. In some cases the incubation period was extended until 36 hours. Any opaque growth observed on the MSRV agar plates was suspected for *Salmonella* and streaked on to Brilliant-green phenol-red lactose sucrose (BPLS) agar (Merck, Germany) by using an inoculating loop which was dipped into the periphery of the opaque zone. Following incubation at 37°C for 24 hours, suspected *Salmonella* colonies were transferred on to 5% blood agar (Blood agar base; Oxoid Ltd., England) plate with incubation for 16 to 18 hours at 37°C. The cultures were identified biochemically and confirmed serologically using anti-*Salmonella* polyvalent serum (SSI, Copenhagen, Denmark). Then all the *Salmonella*-positive cultures were stored at −80°C using 15% glycerol until characterized further.

### Serotyping

All *Salmonella*-positive isolates from the broiler farms, and conveniently selected 21 and 17 isolates from breeder “Farm A” and “Farm B”, respectively with representations from different age groups of the investigated flocks were serotyped using CE (*Conformité Européenne,* placed on products to signify conformance with European Union regulations) marked (ISO) *Salmonella* antisera (SSI Diagnostica, Hillerød, Denmark) and assigned to different serovars according to White-Kauffmann-Le Minor Scheme [3]. To check for autoagglutination, PBS (pH 7.38) was used as a control. Serotyping of the isolates was carried out at Statens Serum Instititut, the National reference laboratory for enteropathogenic bacteria at Copenhagen in Denmark.

### Plasmid Isolation

Plasmid isolation was carried out from all isolates originating from the breeder and the broiler poultry farms following the alkaline denaturation method of Kado and Liu [17], as described by Olsen [18]. One ml overnight shaking culture grown in LB broth at 37°C was used for plasmid isolation. The plasmid DNA was then separated by gel electrophoresis in Tris-acetate EDTA (TAE) buffer on 0.8% agarose (SeaKem® LE agarose, Lonza, Rockland, ME USA) gel at a constant voltage of 120 V for 3 hours. Following electrophoresis, the gel was stained with aqueous solution of ethidium bromide (10 µg/ml; Sigma-Aldrich, USA) and visualized under UV-illumination. The plasmid size was estimated according to references in the literature for *Escherichia coli* V517 [19] and *E. coli* 39R861 [20] and method for size estimation, described by Rochelle *et al*. [21].

### Macrorestriction Analysis by Pulsed-field Gel Electrophoresis (PFGE)

The standardized CDC PulseNet protocol [22] for PFGE was followed to determine the genetic diversity of the isolates representing the breeder- and broiler poultry farms. Restriction enzyme *Xba*I (New England BioLabs Inc.) was used to digest the chromosomal DNA. The chromosomal DNA fragments were isolated using 1% agarose (SeaKem® gold agarose, Lonza, Rockland, ME USA) gel in 0.5× Tris-Borate-EDTA (TBE) buffer with CHEF DR III (Bio-Rad Laboratories, Hercules, California, USA) electrophoresis system at 6 V/cm for 19 hours at 14°C with pulse time switched from 2.2 sec to 63.8 sec under an included angle 120. *Salmonella* Braenderup H9812 was used as a reference size marker [23] in each gel. The gel image was captured by GelDoc EQ system with Quantity One® software (Bio-Rad Laboratories, Hercules, California, USA) and subsequently analysed the macrorestriction DNA fingerprints pattern by GelCompar®II software (Applied Maths, Belgium) version 4.6. The similarity between fingerprints was determined by dice coefficient with a band position tolerance of 1.5% and the unweighted pair group method with arithmetic averages (UPGMA) was applied to generate dendrograms. We followed Tenover’s criteria [24] to interpret the fingerprint patterns of the isolates’ DNA.

## Results

### Farm Status and Distribution of *Salmonella* Serovars

An overview on the flocks observed in the two breeder farms is shown in Table 1. All the flocks in “Farm A” and “Farm B” investigated were *Salmonella*-positive at least for one sampling time. Along with the age progression, not all but four flocks in “Farm A” and three in “Farm B” were intermittently *Salmonella*-positive. A total of 98 isolates - 42 and 56 from “Farm A” and “Farm B”, respectively were obtained over the period of six months. Of them, conveniently selected 38 isolates (21 from “Farm A” and 17 from “Farm B”) of different age groups were serotyped, results of which disclosed the presence of three serovars – *S*. Virchow, *S*. Paratyphi B var Java (*S*. Java) and *S*. Enteritidis (Table 2). *S*. Virchow and *S*. Java were the two predominant serovars identified from both breeder farms (Table 2). One flock in each breeder farm was positive with only one serovar, *S*. Virchow, but the remaining flocks each harboured more than one serovar. One and three flocks in “Farm A” and “Farm B”, respectively, were positive with *S*. Enteritidis plus other serovars.

**Table 1:** Status of motile *Salmonella* in two chicken breeder farms (representing both private and public sectors) in Bangladesh observed for a period of six months, May – October, 2010.

**Farm/Shed No.**	**Age (wk)** *								
	**1–8**	**>8–16**	**>16–24**	**>24–32**	**>32–40**	**>40–48**	**>48–56**	**>56–64**	**>64–72**
╪A/1	–	–	–	–	–	–	–	0	S
A/2	–	–	–	–	–	–	S	S	S
A/7	–	–	–	–	S	S	S	S	–
A/8	–	S	0	S	S	–	–	–	–
A/3	0	S	S	0	–	–	–	–	–
A/4	0	S	S	–	–	–	–	–	–
A/6	–	–	–	–	S	S	S	–	–
A/3(i)	S	–	–	–	–	–	–	–	–
╣B/3B	–	–	–	–	–	S	S	S	–
B/5	–	–	–	S	S	S	0	–	–
B/6	–	–	–	–	–	S	S	S	–
B/7	S	S	–	–	–	–	–	–	–
B/8	S	0	–	–	–	–	–	–	–
B/12	–	–	S	S	S	–	–	–	–
B/3A	–	–	–	–	–	S	S	S	–
B/11	–	–	–	S	S	0	0	–	–

* Frequency of sampling under each age-group = 1–2;

╪ A = A breeder farm under private sector to supply day old broiler chicks to commercial farms;

╣ B* = *A government subsidized breeder farm to produce a cross-bred, “Sonali” (Fayoumi (hen) × RIR (cock)) to supply to the smallholders for being reared in semi-scavenging system; S = *Salmonella*-positive; 0 =  *Salmonella*-negative; –, indicates non-sampling age groups.

**Table 2:** Distribution of *Salmonella* serovars from breeder- and commercial broiler poultry farms in Bangladesh, 2010.

Farm category	No. isolates serotyped	Distribution of *Salmonella* serovars (n)
Breeder Farm A*	21	Virchow (11)
		Paratyphi B var Java (9)
		Enteritidis (1)
Breeder Farm B**	17	Paratyphi B var Java (7)
		Virchow (6)
		Enteritidis (4)
Broiler Farm	11	Virchow (5)
		Kentucky (4)
		Paratyphi B var Java (1)
		Weltevreden (1)

* A broiler breeder farm in commercial poultry sector in Bangladesh.

** A breeder farm in public poultry sector in Bangladesh to produce F1 generation (locally named ‘Sonali’) (Fayoumi (Hen) and Rhode Island Red (Cock)).

The cross-sectional survey revealed that 11 out of the 100 broiler poultry farms were *Salmonella*-positive in one or more of the five samples collected, giving a prevalence of 11% (95% confidence interval 5–17%) at farm level. *S.* Virchow was the predominant serovar followed by *S.* Kentucky (Table 2). Two other serovars – *S.* Java (n = 1) and *S.* Weltevreden (n = 1) were also isolated from two broiler farms.

### Plasmid Profiles

All the *S.* Virchow and *S.* Java isolates from “Farm A” and “Farm B” were plasmid-free, whereas the *S.* Enteritidis isolates carried an approximately 60 kb plasmid. Twenty out of the 21, and 35 out of the 39 non-serotyped isolates from “Farm A” and “Farm B”, respectively, were plasmid-free, but each of the remaining ones had a 60 kb-sized plasmid, similar to that seen in the *S.* Enteritidis isolates.

**Figure 1:**
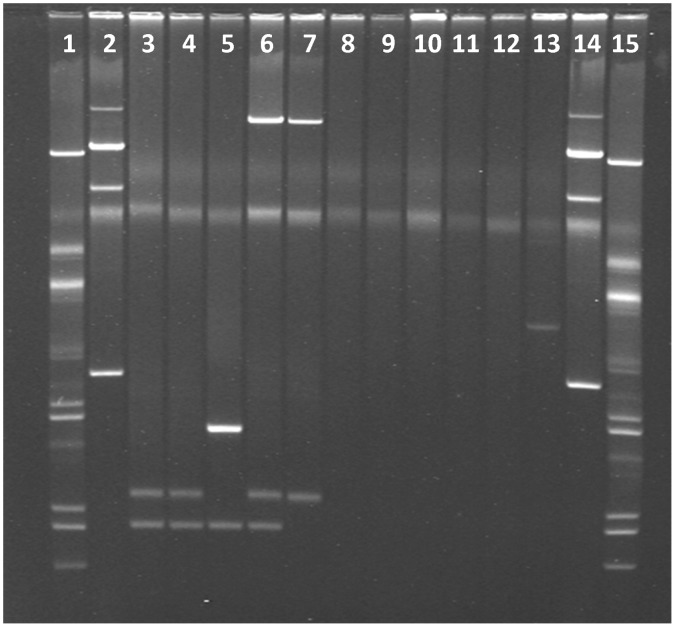
Plasmid profiles of 11 *Salmonella* isolates belonging to four different serovars, from commercial broiler poultry farms in Bangladesh, May – July 2010. (Lanes 3–6 for *S*. Kentucky; Lane 7 for *S*. Weltevreden; Lane 8 for *S*. Paratyphi B var Java, Lanes 9 - 13 for *S*. Virchow, Lanes 1 and 15, and Lanes 2 and 14 are plasmid size markers in *Escherichia coli* strains V517 and 39R861, respectively).

The plasmid profiles of the broiler farms’ isolates are shown in Figure 1. Four different sized plasmids displaying three distinctive profiles were observed in the *S.* Kentucky isolates. Two small plasmids of approximately 2.7 and 3.5 kb were common to all of them, except for one that although contained two pieces, their sizes were of approximately 2.7 and 4.8 kb. One isolate had an approximately 109 kb-sized plasmid together with two smaller ones of 2.7 and 3.5 kb. Like breeder farms, *S*. Java and *S*. Virchow isolates (n = 4) from broiler farms were also plasmid-free, with a single exception of one *S*. Virchow isolate, that contained an approximately 9.7 kb plasmid. The *S.* Weltevreden isolate harboured two plasmids of approximately 3.5 and 109 kb sizes.

### Genotyping by PFGE

**Figure 2:**
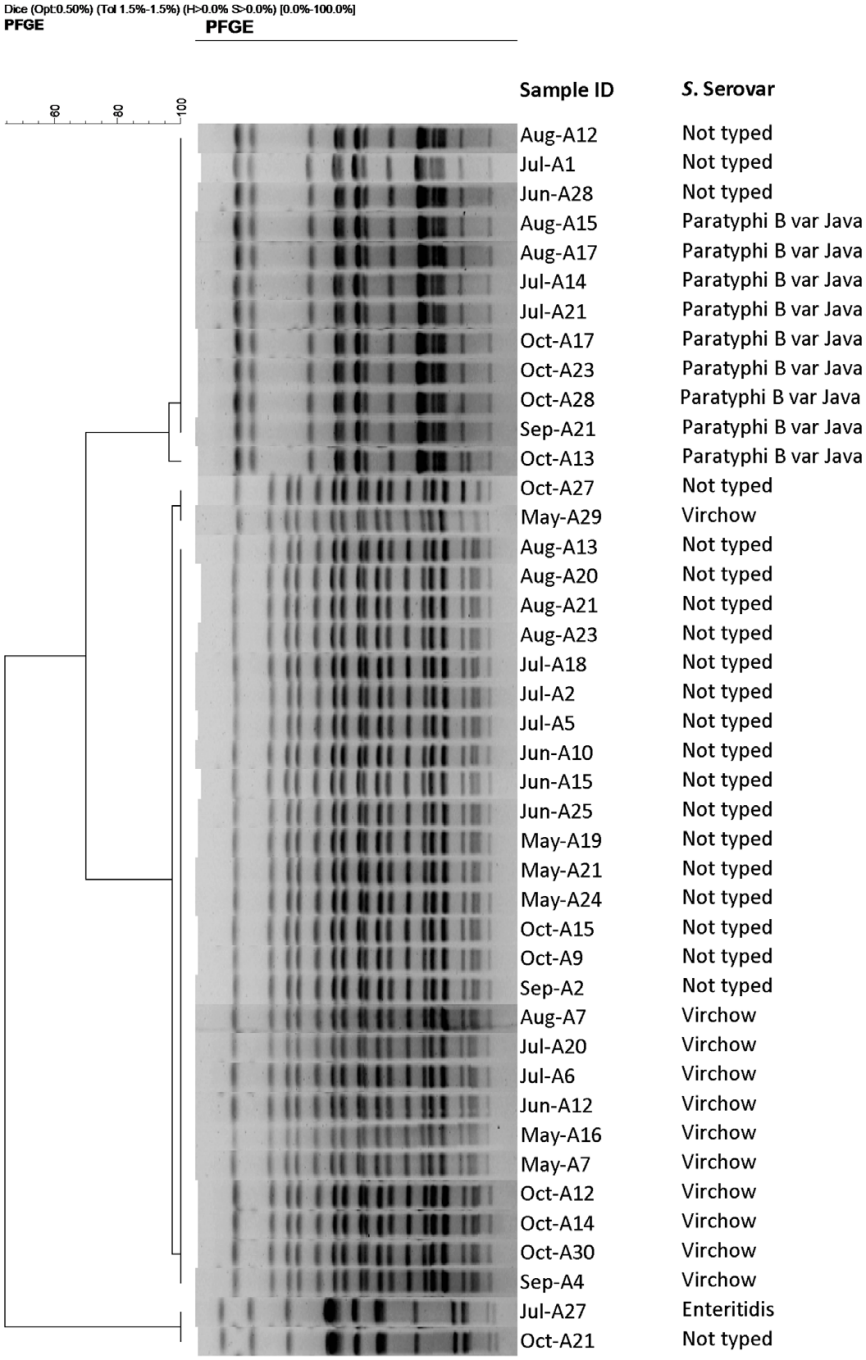
Dendrogram showing the cluster analysis on the basis of *Xba*I-PFGE of the *Salmonella* isolates belonging to different serovars and non-serotyped isolates, obtained from a broiler breeder farm (in the text referred to as “Farm A”) in the commercial poultry sector in Bangladesh, May – October, 2010. Dice coefficient was used to perform similarity analysis, and clustering was performed by using unweighted pair-group method with arithmetic means (UPGMA) with 1.5% band position tolerance.

**Figure 3:**
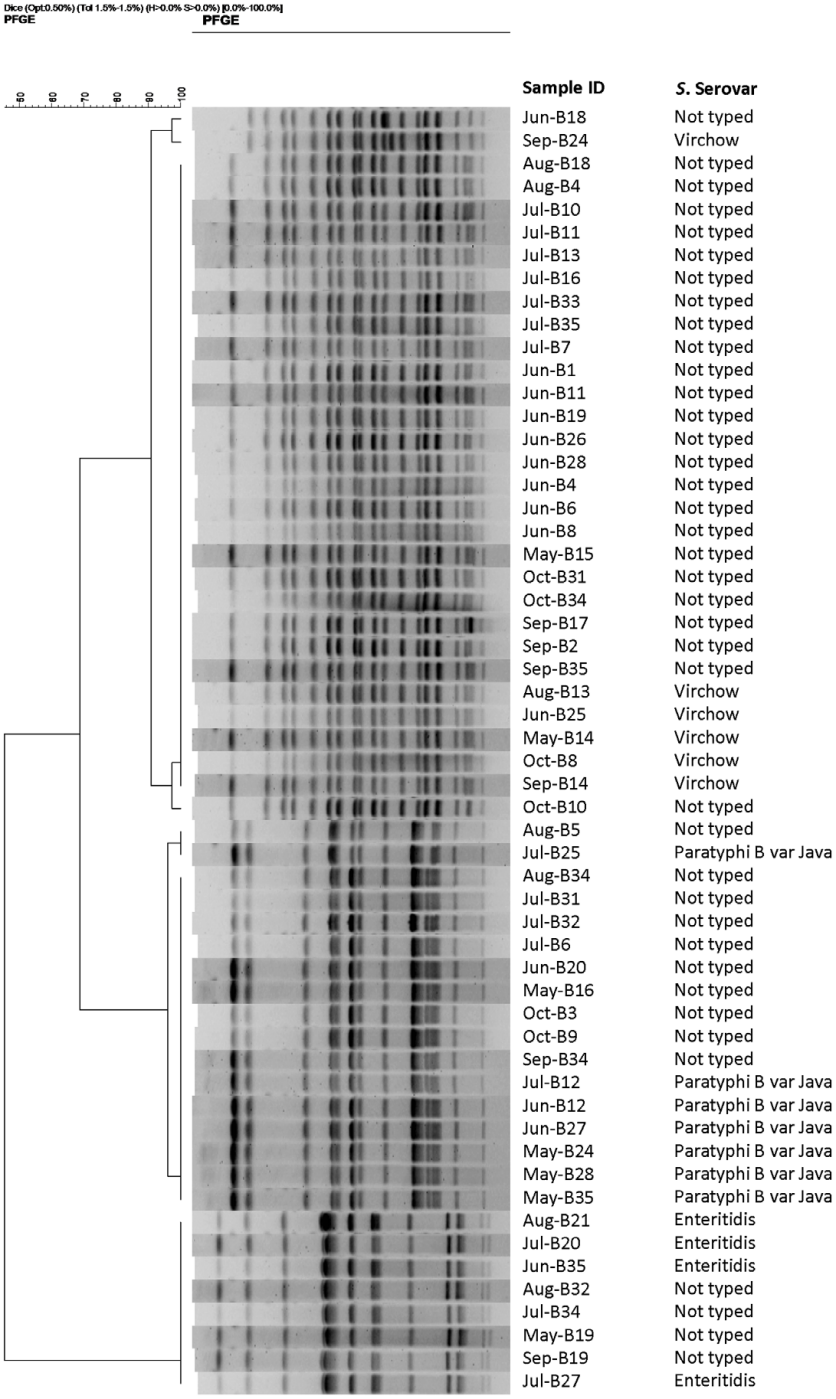
Dendrogram showing the cluster analysis on the basis of *Xba*I-PFGE of the *Salmonella* isolates belonging to different serovars and non-serotyped isolates, obtained from a breeder farm in the public poultry sector (in the text referred to as “Farm B”) in Bangladesh to produce F1 generation (locally named ‘Sonali’) (Fayoumi (Hen)× Rhode Island Red (Cock)), May – October, 2010. Dice coefficient was used to perform similarity analysis, and clustering was performed by using unweighted pair-group method with arithmetic means (UPGMA) with 1.5% band position tolerance.

**Figure 4:**
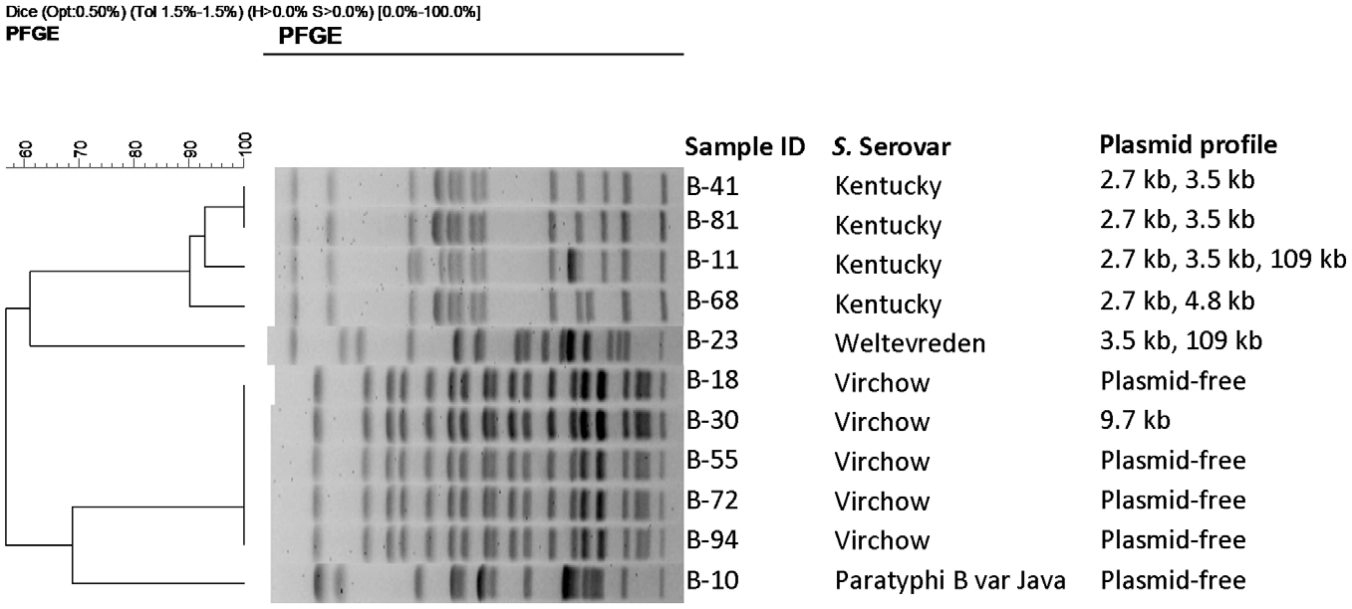
Dendrogram showing the cluster analysis on the basis of *Xba*I-PFGE of the 11 *Salmonella* isolates belonging to four serovars (*S*. Kentucky = 4, *S*. Virchow = 5, *S*. Paratyphi B var Java = 1 and *S*. Weltevreden = 1) obtained from a cross-sectional survey in commercial broiler poultry farms in Bangladesh, May – July, 2010. Dice coefficient was used to perform similarity analysis, and clustering was performed by using unweighted pair-group method with arithmetic means (UPGMA) with 1.5% band position tolerance.

The dendrograms illustrating the cluster analyses of the isolates from “Farm A”, “Farm B”, and the broiler poultry farms are shown in Figures 2, 3 and 4, respectively. Based on variations in one to four bands, two closely related pulsotypes were seen among the *S*. Virchow isolates, originating from both breeder farms. By single band difference, two closely relating fingerprint patterns were also identified in the *S*. Java isolates from “Farm A” (n = 9) and “Farm B” (n = 7). Owing to cent per cent band-homogeneity, all the *S.* Enteritidis isolates were clonal. Of the 21 non-serotyped isolates from “Farm A”, 17 and 3 isolates showed similar band patterns to that of the *S*. Virchow and *S*. Java isolates, respectively. Out of the 39 non-serotyped isolates from “Farm B”, *S*. Virchow like band-pattern was observed in 25 isolates and *S*. Java like in 10. The non-serotyped isolates (1 in “Farm A” and 4 in “Farm B”) that showed identical plasmid profile to those of the *S*. Enteritidis isolates also had a *S*. Enteritidis isolate like band pattern. By differences in ≥2 bands, *S.* Kentucky isolates formed three closely relating genotypes.

## Discussion

In both breeder farms, some flocks were persistently and some were intermittently *Salmonella*-positive, suggesting the continuing presence of *Salmonella* in the farms and/or environment [25]. Serotyping further revealed that, probably, three zoonotic *Salmonella* serovars: *S*. Virchow, *S*. Java and *S*. Enteritidis are circulating in the breeder farm environments in Bangladesh. It is remarkable that the same serovars are prevalent in the commercial and public sector farms and it clearly indicates a common source for the colonisation. The distribution of *Salmonella* serovars in the broiler poultry farms, however, was more diverse as two other serovars: *S.* Kentucky and *S.* Weltevreden in addition to *S.* Virchow and *S.* Java were demonstrated from the broilers. To the authors’ knowledge, this is also the first report on specific motile *Salmonella enterica* serovars in breeder and broiler poultry farms in Bangladesh.

The two breeder farms investigated were located about 50 km apart from one another, and the circulation of the same serovars with closely related genotypes suggests common sources to the infections, but this was not investigated further in this study. Some of the raw feed ingredients might be a commonality for both breeder farms. Moreover, porous biosecurity and same ecological factors might also pose a risk for infection. The results of serovar distribution and subsequently genotyping of both serotyped and non-serotyped isolates by PFGE also demonstrated that multiple flocks in the same breeder farm which harboured a single serovar at an early age were positive with 1–2 other serovars over the course of time. The presence of the same serovar, particularly *S*. Virchow in multiple flocks could be due to the horizontal transmission from the hatchery or vertical transmission and subsequently infection with other serovars could originate from e.g. contaminated farm environment or feed stuff [26]. Occurrence of *S*. Enteritidis in some flocks at an early age suggests the vertical transmission of this serovar [27].

One of the major findings of the cross-sectional survey of the broiler farms is the presence of *S*. Kentucky (Table 2), the only serovar found in layer poultry farms in Bangladesh in a previous comprehensive survey [15]. Thus its circulation in both the commercial broiler and the layer production systems in Bangladesh might pose a potential threat to the public health [9], for consequently being transmitted to humans from both poultry meat and eggs. Poultry is considered a major reservoir for *S*. Kentucky [10]. Its circulation in broiler flocks [28], [29], and zoonotic role has previously been reported [9]. Because the serovar was not isolated from any of the breeder farms they are not considered to be the main source in Bangladesh. However, there are some reports indicating protein concentrates in animal feed as sources for *S*. Kentucky [30]. Whether raw dry-fish ingredients used as protein sources to formulate poultry feed in the study area, as hypothesized in our previous study [15], are a source for *S.* Kentucky or not, should be investigated in future epidemiological studies.

All the sampled flocks in both breeder farms were *Salmonella*-positive at least in one sampling (Table 1). A lower carriage proportion in the population and/or intermitting shedding phenomenon from the infected chickens [31] might explain why some flocks were *Salmonella*-negative in a particular month, having diagnosed positive in the preceding month (Table 1).

We acknowledge our limitation that we did not serotype all the isolates obtained from the breeder farms. A subset of isolates isolated from different age groups were conveniently selected and subsequently serotyped. However, we characterized all the isolates by plasmid profiling and PFGE, results of which demonstrated that the non-serotyped isolates displayed similar plasmid and PFGE profiles to the serotyped ones (Figures 2 and 3), strongly suggesting that these isolates could also be classified any of the three serovars identified.

The prevalence of motile *Salmonella* in commercial broiler poultry farms was 11%, lower than the 18%, reported from layer poultry farms in the same study area [15]. Although the sample size of broiler farms investigated in the cross-sectional survey part was small, the results are highly useful as it is the first time that the prevalence of motile *Salmonella* at small-scale commercial broiler farm level is demonstrated from Bangladesh; however, variations in prevalence have been reported from many other geo-locations [32]–[34].

Unlike *S*. Kentucky, *S.* Virchow was found in both breeder and broiler poultry farms and its isolation frequency was the highest in Farm A and in the broiler farms (Table 2). This poultry associated serovar has been reported from the broilers and breeder flocks from the member states of the European Union (EU) [35]. *S*. Virchow has also been isolated from chickens in Thailand [36] and broiler farms in Algeria [34]. *S.* Virchow constitutes one of the five EU-top prioritised serovars to be investigated owing to its public health significance [32]. *S.* Java was frequently isolated from the breeder farms, although a single isolate was obtained from a broiler farm. This is a recently emerging serovar reported particularly from the Netherlands [7], [37], Germany [38] and imported poultry meat has been identified its major source to infect humans in Scotland [39]. Single or multiple flocks in each breeder farm were also positive with *S.* Enteritidis, which is alarming, because this is the only serovar that truly infects the oviduct of poultry, thereby being transmitted to their progeny vertically [40] or pseudo-vertically. *S*. Weltevreden was isolated from one broiler farm, a rarely reported serovar from chickens [36], but commonly has been isolated from humans in Thailand [8].

Plasmid profiling is one of the applied molecular tools for subtyping *Salmonella* [41]. Plasmid free isolates of *S*. Virchow and *S*. Java were identified in breeder and broiler farms, except for one *S*. Virchow isolate that harboured a single plasmid. Plasmid free and single to multiple plasmid containing *S*. Java has been described before [39]. The *S*. Enteritidis isolates contained only one plasmid of 60 kb, in agreement with the findings of Kalender *et al*. [42] who also reported multiple plasmid containing isolates in Turkey. The *S.* Kentucky isolates displayed three distinct plasmid profiles among which one profile (2.7 kb, 4.8 kb) is similar to the isolates previously described from layer poultry farms in Bangladesh [15]. One isolate harboured a large plasmid (109 kb), which differs with the findings of Majtán *et al*. [43] who documented two large plasmids (40 kb, 90 kb) from human isolates in Slovak Republic.

The PFGE results demonstrated that the two serovars – *S*. Virchow and *S*. Java, isolated from the breeder poultry farms comprise of two closely relating pulsotypes, however, all *S*. Virchow isolates from the broiler poultry farms were clonal. The broiler clone was also present in “Farm A”. Three closely related genotypes of *S*. Kentucky are circulating in the broiler farms, one of them was identical to a *S*. Kentucky genotype previously identified from layer farms in the same study area [15]. All the *S*. Enteritidis isolates were clonal. One and four non-serotyped isolates from “Farm A” and “Farm B”, respectively, displayed the similar plasmid (60 kb) and PFGE profiles (Figures 2 and 3) to those of *S*. Enteritidis, and therefore, it was speculated that, they probably belonged to the same *S*. Enteritidis clone.

In conclusion, the investigated breeder poultry farms in Bangladesh were persistently harbouring at least three zoonotic serovars, namely *S*. Virchow, *S*. Java and *S*. Enteritidis; the first two were also common in broiler poultry farms in addition to two others – *S*. Kentucky and *S.* Weltevreden. The prevalence of motile *Salmonella* at broiler farm level was 11% and at least one of the three genotypes of *S.* Kentucky, unveiled previously from commercial layer poultry farms in the same study area, is also circulating in broiler farms. *S*. Virchow and *S*. Java each has two closely relating pulsotypes, whereas one clone of *S*. Enteritidis has established itself in breeder farms. Further molecular epidemiological studies are needed to identify the sources of infection, and to explore the impact of this zoonosis on public health in Bangladesh and beyond.
